# Quantitation of paracetamol by liquid chromatography–mass spectrometry in human plasma in support of clinical trial

**DOI:** 10.4155/fsoa-2018-0039

**Published:** 2018-08-15

**Authors:** Richard Kin-Ting Kam, Michael Ho-Ming Chan, Hiu-Ting Wong, Aniruddha Ghose, Arjen M Dondorp, Katherine Plewes, Joel Tarning

**Affiliations:** 1 Department of Chemical Pathology, Prince of Wales Hospital, The Chinese University of Hong Kong, Hong Kong SAR, PR China; 2 Department of Medicine, Chittagong Medical College Hospital, Chittagong, 4217, Bangladesh; 3 Nuffield Department of Clinical Medicine, Centre for Tropical Medicine & Global Health, University of Oxford, Old Road campus, Roosevelt Drive, Headington, Oxford, OX3 7FZ, UK; 4 Mahidol-Oxford Tropical Medicine Research Unit, Faculty of Tropical Medicine, Mahidol University, 420/6 Ratchawithi Road, Rajthevee, Bangkok, 10400, Thailand

**Keywords:** acetaminophen, bioanalysis, LC–MS/MS, malaria, paracetamol

## Abstract

**Aim:**

Paracetamol is a well-tolerated antipyretic widely used in severe malaria management. The study aimed to develop and validate a rapid LC–MS/MS assay to quantify paracetamol in plasma from patients with severe malaria.

**Materials & methods:**

Plasma sample was precipitated by organic solvent containing isotope-labeled paracetamol internal standard. Supernatant was isolated, diluted with water, followed by LC–MS/MS analysis.

**Results:**

Plasma samples were extracted and assayed in less than 5.5 min. The assay response was linear (0.125–50 mg/l) with total intra- and interassay imprecision of <1.4%, which were considerably lower than most published reports.

**Conclusion:**

We developed, validated and applied a rapid and small volume LC–MS/MS assay with high precision and accuracy for plasma paracetamol quantitation in 989 samples from 62 patients with severe malaria. The simple and high-throughput quality could facilitate assay automation for future clinical studies.

## Lay abstract

Paracetamol is a commonly prescribed antipyretic in severe malaria management. Here, a rapid quantitative assay was developed to measure the plasma level of paracetamol. The developed method required levels as low as 20 μl plasma, and was highly precise, with a short analysis time of 5.5 min. The developed method is particularly suitable for clinical trial application and potentially for clinical use.

Paracetamol is a common over-the-counter, nonopioid and nonsteroidal analgesic listed as an essential medicine by the World Health Organization [1]. Since its discovery, numerous studies have reported on the analgesic and antipyretic effects of paracetamol [2–5]. While paracetamol was found to be much less toxic than its precursor drugs, acetanilide and phenacetin, there is potential risk of hepatotoxicity related to overdose [6,7]. In recent years, additional clinical effects of paracetamol have been studied including the reduction of oxidative stress-mediated acute kidney injury [8,9], management of patent ductus arteriosus in preterm infants [10,11] and the association with reduced mortality as an adjunct to antibiotics in pediatric meningitis [12].

In routine clinical laboratories, paracetamol was previously quantified using an arylacylamidase enzymatic method followed by colorimetry [13]. However, this method is subject to interference with bilirubin, rendering the test inaccurate for quantitation in samples from patients with liver dysfunction and/or hyperbilirubinemia [14,15]. Other methodologies for paracetamol quantification have since been developed including gas chromatography [16], GC–MS [17–19], and reverse-phase HPLC [20,21]. However, quantitation of paracetamol using LC–MS/MS is considered a more specific method [22]. Many reports have described and validated different LC–MS/MS-based methodologies to quantitate paracetamol in different sample matrices [23], some with simultaneous metabolite quantitation [22,24–29].

In severe falciparum malaria, intravascular hemolysis results in elevated concentrations of bilirubin and cell-free hemoglobin [30]. Redox cycling of cell-free hemoglobin can cause pathologic oxidative stress [31,32]. Paracetamol has been shown to exert a protective effect by reducing heme-protein-induced lipid peroxidation [9,33–35]. A randomized control trial (RCT) of paracetamol was conducted in severe and moderately severe malaria patients to assess its potential renoprotective effect via reducing cell-free hemoglobin mediated kidney injury [36]. Quantitation of plasma paracetamol concentrations was planned as part of this RCT to determine the pharmacokinetic properties and dose-response relationship of paracetamol in this study population. Thus, a sensitive methodology was required to quantify paracetamol in this population, given that both bilirubin and hemoglobin may interfere with paracetamol quantification [37].

Here we report a simple and rapid LC–MS/MS method for paracetamol quantitation using as little as 20 μl of plasma. The method described had lower imprecision and shorter run-time compared with most reported LC–MS/MS assays. As a demonstration of its application, the assay was used to quantitate paracetamol concentrations in patient samples from a RCT of paracetamol in severe and moderately severe malaria (ClinicalTrials.gov registration number: NCT01641289).

## Materials & methods

### Reagents

Pharmaceutical standard of paracetamol and analytical standard of paracetamol-(ring-D_4_) were purchased from Sigma–Aldrich (MO, USA). Plasticware was purchased from Eppendorf (Hamburg, Germany). Anonymous drug-free EDTA plasma was pooled from patient samples in the Department of Chemical Pathology, Prince of Wales Hospital, Shatin, Hong Kong.

A stock solution of 200 mg/l paracetamol was prepared in HPLC grade methanol. A working solution of 100 mg/l was prepared by mixing equal volumes of stock solution and HPLC grade methanol in a microcentrifuge tube. Both the stock and working solutions were stored at -80°C before use.

A stock solution of 200 mg/l paracetamol-D4 was prepared in HPLC grade methanol in a 1.5 ml eppendorf. A working internal standard solution at 200 μg/l was prepared by diluting the stock solution in 100 ml HPLC grade methanol. The working internal standard was stored at 4°C.

### Calibration standards and quality control samples

A 6-level series of calibrators was prepared using drug-free pooled plasma. Briefly, pooled plasma was spiked with stock paracetamol solution to prepare 50, 16, 4, 1, 0.25 and 0.125 mg/l calibrators. Pooled plasma was used as blank.

A 3-level quality control (QC) series of samples were prepared using pooled plasma (final percentage of methanol = 25% v/v). Briefly, pooled plasma was spiked with working paracetamol solution to prepare 30, 5 and 0.25 mg/l QC samples. An extra level of 0.125 mg/l was prepared for validation purposes as the lower limit of quantitation (LLOQ). All calibration standards and QC samples were aliquot and stored at -80°C for storage.

### Sample preparation

Clinical plasma samples, QC and calibrators were thawed at room temperature for 15 min on a rotary mixer at room temperature prior to processing. Briefly, 20 μl of sample, QC or calibrators were mixed with 320 μl of working internal standard solution in a 1.5 ml microcentrifuge tube, followed by vortex mixing for 5 min and centrifugation at 17,000 × g for 5 min. Subsequently, 20 μl of supernatant was diluted 50-fold using MilliQ water. 10 μl of the diluted sample was then subjected to LC–MS/MS analysis. For method validation purposes, blank matrix was prepared from drug-free plasma, with working internal standard solution replaced by pure HPLC-grade methanol.

### Instrumentation

The LC–MS/MS system consisted of a Waters I-Class UPLC system coupled to Waters Xevo TQ-S (MA, USA). Paracetamol was separated from plasma matrix on a Waters ACQUITY BEH C18 50 × 2.1 mm, 1.7 μm UPLC column (MA, USA) at a flow rate of 0.3 ml/min. A gradient mobile phase was used; starting at 95% mobile phase A (0.1% formic acid in MilliQ water) and 5% mobile phase B (100% HPLC grade methanol), with a linear increase over 3.5 min to 35% mobile phase B, followed by 1 min of washing at 95% mobile phase B and 1 min re-equilibration to initial condition. The total run time was 5.5 min. Paracetamol and internal standard paracetamol-D4 were eluted at 2.25 ± 0.05 min and monitored by multiple reaction monitoring (MRM) transition *m/z* 152 > 110 and *m/z* 156 > 114, respectively. An extra MRM transition 152 > 65 was used for paracetamol as qualifier.

LC eluate was sprayed into Waters Xevo TQ-S mass spectrometer by electrospray ionization in the positive ion mode. Capillary voltage was 2.3 kV with cone voltage at 40 V. Ion source temperature was maintained at 150°C with desolvation temperature at 600°C. The LC–MS/MS instrument was controlled by using Waters MassLynx software (version 4.1, MA, USA).

### Data analysis

Data acquired was processed using Waters TargetLynx software (version 4.1). Signal response was calculated as the ratio between the LC peak areas of paracetamol to that of the internal standard. The calibration curve was plotted using 6-level calibrators and a blank. The US FDA Guidance for Industry Bioanalytical Method Validation (2013) states that the simplest model that adequately describes the concentration–response relationship should be used [38]. Thus, different calibration curves were evaluated for each batch of analysis by using log–log, quadratic and linear regression with and without weighting (in other words, 1 and 1/x). The calibration curves were evaluated based on the overall performance as described previously [39]. A maximum relative bias of ± 15% and ± 20% was allowed for calibration points and for LLOQ, respectively. Relative bias was calculated as follows: relative bias = 100 × (observed concentration - nominal concentration)/nominal concentration.

### Method validation

The described method was validated for linearity, imprecision, inaccuracy, recovery, matrix effect, stability and carryover.

### Method validation: linearity

The calibration curve from 0.125 to 50 mg/l was analyzed over 3 days for the assessment of accuracy and precision. Relative bias from a nominal concentration within 15 (20% for LLOQ), was defined as an acceptable criteria. The different calibration models were evaluated based on their relative bias in back-calculated calibrators and predictability of QC samples according to Singtoroj *et al*. 2006 [39]. Briefly, the calibration curve fit and QC imprecision and accuracy were ranked for all regression models tested. The rank sum was then used to assess the overall performance of regression models. Data from seven batches of analysis were used for the evaluation.

### Method validation: imprecision & inaccuracy

Intra- and inter-assay imprecision was assessed by coefficient of variation (CV%), while inaccuracy was assessed by relative bias. For intra-assay imprecision, five sets of 3-level QC and LLOQ samples were analyzed in a single batch of analysis. For inter-assay imprecision, three sets of 3-level QC and LLOQ samples were analyzed over three days. CV% within 15 (20% for LLOQ), was defined as acceptable criteria. Similarly, deviations from nominal concentrations within 15 (20% for LLOQ) were defined as an acceptable criteria for inaccuracy.

### Method validation: process efficiency, extraction recovery & matrix effect

Process efficiency was assessed by comparing the observed concentration of each sample with the equivalent concentration of pure aqueous standard. Extraction recovery was assessed by comparing the observed concentration of spiked plasma samples to that of the extracted blank spiked with an equivalent amount of analyte. Potential matrix effects of the method were assessed by both post-column infusion of 1 mg/l paracetamol and paracetamol-D4 into the ion-source over the LC–MS/MS analysis of blank matrix, and the spike-recovery method. For the post-column infusion method, the matrix effect was visualized and also calculated using Waters TargetLynx Matrix Calculator. For spike-recovery method, the observed concentration of the extracted blank spiked with analytes was compared with equivalent concentration of pure aqueous standard.

The three parameters were derived mathematically according to Equations 1–3.(1)
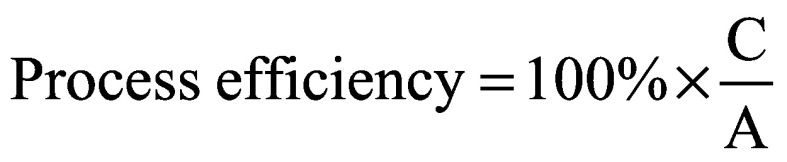

(2)
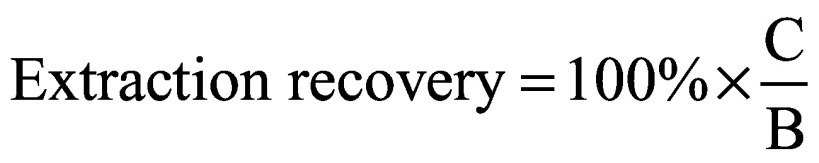

(3)
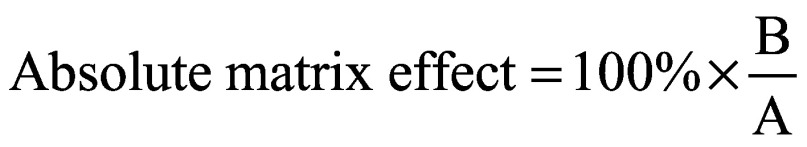



where A = standard prepared in reconstitution solvent; B = standard spiked to the extracted blank matrix; C = standard spiked to plasma followed by sample extraction.

### Method validation: carryover

Carryover was assessed based on the method described by Zeng *et al*. [40]. Briefly, a sample at the upper limit of quantitation (ULOQ) of 50 mg/l was extracted and analyzed by LC–MS/MS, followed by an injection of MilliQ solvent blank. The relative carryover between samples was calculated as the observed peak area in solvent blank relative to that of the previous injection of high concentration sample. The maximum allowable concentration ratio between consecutive injections was defined as the ratio of concentration between injection, in which the carryover of the first injection contributes significantly (>5%) to the second injection. Maximum allowable concentration ratio was derived as 5% relative carryover.

### Clinical applicability

Clinical applicability was demonstrated by quantitation of paracetamol in plasma samples from patients with malaria. Patient samples were provided by the Mahidol-Oxford Tropical Medicine Research Unit (MORU), Faculty of Tropical Medicine, Mahidol University (Bangkok, Thailand). Paracetamol (1 g) was administered enterally every 6 h for 72 h to patients with severe and moderately severe *P lasmodium falciparum* (*P. falciparum*) malaria.

## Results & discussion

**Figure 1.**
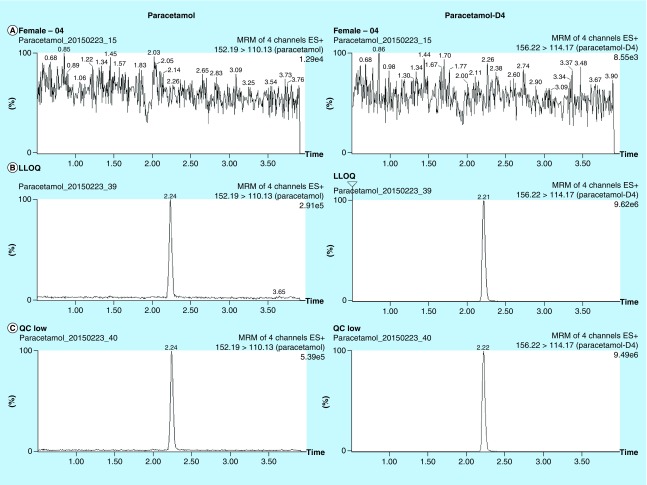

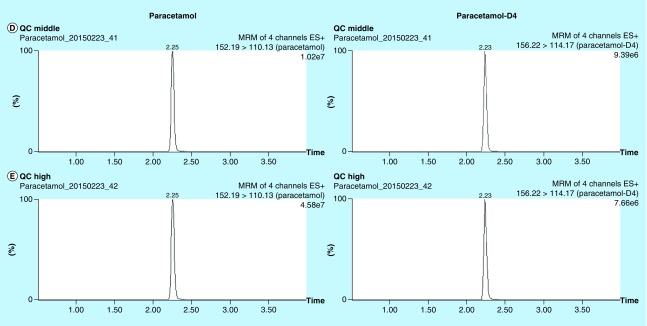
**Extracted ion chromatograms of paracetamol (left column) and paracetamol-D4 (right column).** **(A)** Plasma blank, **(B–E)** QC samples spiked with paracetamol at **(B)** 0.125 mg/l (LLOQ), **(C)** 0.25 mg/l, **(D)** 5 mg/l and **(E)** 30 mg/l. Extracted ion chromatograms of paracetamol-D4 represented post-extraction concentration of 3.8 mg/l. LLOQ: Lower limit of quantitation; QC: Quality control.

The aim of the current method was to develop and validate a bioanalytical assay for paracetamol with a simple sample preparation, short LC–MS/MS run time, and high precision and accuracy. A small volume of plasma sample was precipitated with methanol containing internal standard followed by 50-fold dilution, which substantially reduced matrix interference and minimized the handling step and within-batch imprecision. Organic solvent precipitation also facilitated paracetamol solubilization and denaturation of plasma binding protein. These factors were reflected by the quantitative recovery of paracetamol from plasma and the absence of matrix effect. The reduction of matrix effect allowed the use of a rapid LC program of 5.5 min per run. Since the procedure only involved protein precipitation with solvent dilution, the method can be automated by liquid handler with 96-well plate which would increase the throughput of analysis. The typical mass chromatogram of paracetamol and paracetamol-D4 in plasma is shown in Figure 1. The S/N ratio was >10 at all concentrations within the linear range (0.125–50 mg/l). The current method was developed and validated according to the principle of FDA Guidance for Industry, Bioanalytical Method Validation, 2013, which stated the use of six nonzero samples covering the expected ranges including LLOQ. We believed the performance of calibration curves can adequately demonstrate the linear concentration-response relationship of the method within the expected ranges.

By using the optimized LC gradient, paracetamol was eluted at 2.25 ± 0.05 min, while paracetamol-D4 eluted slightly earlier, a phenomenon commonly observed for deuterium-labeled compounds [41]. The total run-time was 5.5 min with column washing and re-equilibration included. The chromatographic baseline was steady and consistent in the LC–MS/MS run, due to the extensive dilution of precipitated plasma by water. As reflected by matrix effect evaluation, endogenous interference was negligible. The run-time and sample volume was lower than other previously published methods (Table 2).

### Accuracy & precision

**Figure 2.**
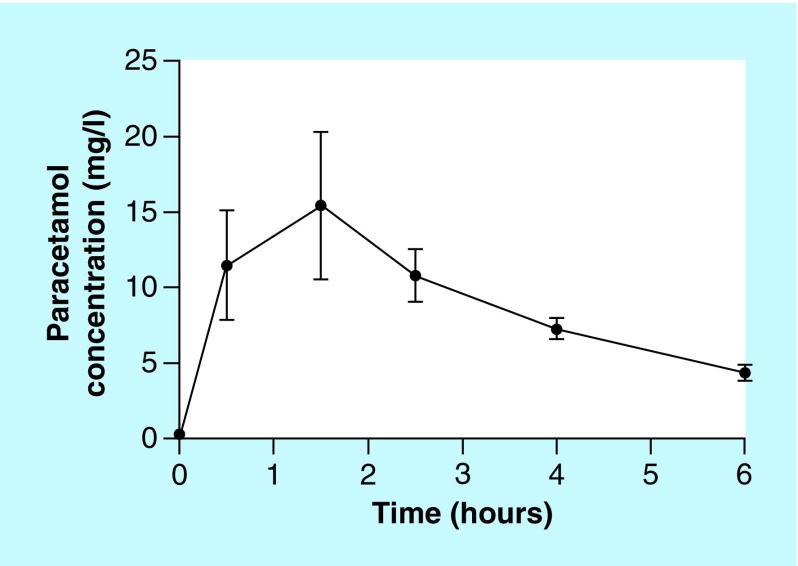
**Measured concentration–time profiles of paracetamol in patients with malaria, after a single oral dose of 1 g paracetamol (n = 5).**

Simple methanol precipitation was chosen as the sample preparation strategy since it was less laborious compared with liquid–liquid extraction or SPE. At LLOQ (0.125 mg/l) and QC levels (0.25, 5 and 30 mg/l), we achieved intra-assay imprecision less than 1.8% CV (1.8, 0.9, 1.0 and 1.0%, respectively; Table 1), and inter-assay imprecision less than 2.3% CV (2.3, 0.9, 0.7 and 1.4%, respectively; Table 1). Compared with the nominal value, the relative bias at all concentrations was less than 15%. The imprecision was comparatively smaller than other published reports (Table 2), which commonly reported intra-assay CV% of approximately 2.8–13%. If LLOQ was excluded, the achieved imprecision in this study was <1 and <1.4%, respectively (Table 2). Since the analytical variation was relatively small, the variation observed in plasma would more precisely reflect the biological variation associated with the patient samples and collection procedures (Figure 2). We also assessed long-term imprecision from 13 batches of analysis over 1 month, and found that the overall imprecision at all levels was <2%. By reducing the manual procedure during sample preparation, more than 150 clinical samples were analyzed per day manually. It was also possible to migrate the current method to an automated liquid handler, and further increase the throughput of the assay.

**Table 1.** **Intra-assay, interassay imprecision and inaccuracy of plasma paracetamol quantitation.**

**Nominal concentration (mg/l)**	**Observed concentration (mg/l)**	**Intra-assay**			**Observed concentration (mg/l)**	**Inter-assay**		

		**Mean accuracy (%)**	**SD**	**%CV**		**Mean accuracy (%)**	**SD**	**%CV**
0.125	0.122	86.6	2.0	1.8	0.122	86.3	2.5	2.3

0.119				0.117				

0.119				0.123				

0.123								

0.120								

0.25	0.251	98.9	2.3	0.9	0.251	99.1	2.3	0.9

0.254				0.250				

0.250				0.255				

0.253								

0.249								

5	5.282	110.0	55.4	1.0	5.282	110.1	40.0	0.7

5.226				5.224				

5.290				5.180				

5.215								

5.161								

30	32.920	109.4	334.5	1.0	32.920	109.3	462.9	1.4

32.465				31.978				

32.656				32.327				

32.086								

32.151							*	

%CV: Coefficient of variation.

**Table 2.** **Summary on the analytical performance of published plasma paracetamol LC–MS/MS quantitation methods.**

**Year of study**	**Method description**	**Sample volume (μl)**	**LLOQ (mg/l)**	**LC run-time (min)**	**Linearity (mg/l)**	**%CV (exclude LLOQ)**		**Ref.**

						**Intra-assay**	**Interassay**	
2017	Protein precipitation	20	0.125	5.5	0.125–50	<1.0	<1.4	Current study

2015	Protein precipitation, vacuum drying and reconstitution	10	0.05	20	0.05–50	<7.1%	<7.2%	[26]

2013	Protein precipitation	50	0.003	4.2	0.003–20	<4.9	<8.7	[23]

2013	Protein precipitation, vacuum drying and reconstitution	25	0.25	10	0.25–20	<2.9	<6.3%	[25]

2012	Protein precipitation, vacuum drying and reconstitution	50	0.020	18	0.02–10	<3.9%^†^		[24]

2011	Protein precipitation	100	0.03	NA	0.03–9	<8.4%^†^		[43]

2010	Protein precipitation	50	0.01	6	0.01–5	<6.8	<10.0	[28]

2008	Protein precipitation	50	0.012	6	0.012–25	<10.8	<13.0	[44]

2006	Protein precipitation, followed by SPE	100	5	70	5–100	<5.7	<6.3	[45]

2003	Protein precipitation	NA	0.01	25	0.01–5	<3.4%^†^		[46]

^†^Not specified.

LLOQ: Lower limit of quantitation; NA: Not available.

The method developed was intended to support randomized clinical trial (RCT) on the renoprotective effect of paracetamol on reducing cell-free hemoglobin mediated kidney injury. In the bioanalysis of submitted samples (n = 989), 330 samples were distributed between high (30 mg/l) and middle (5 mg/l) level QC; 337 samples were between middle and low (0.25 mg/l) level QC; 40 samples were between low level QC and LLOQ (0.125 mg/l). Only two samples were above the high level QC concentration. The remaining samples were predose samples with undetectable paracetamol. Overall, the selected QC concentrations were able to cover the observed concentration in bioanalytical samples.

Incurred sample reanalysis (ISR) was conducted according to FDA Guidance 2013 for the RCT samples submitted. A total of 81 samples were selected for ISR, and 79 samples (97.5%) were within the acceptance criteria of ISR (± 20% deviation).

### Process efficiency, extraction recovery & matrix effect

The spike-recovery study indicated a complete, reproducible and consistent recovery of paracetamol in plasma over different concentrations. The overall process efficiency was estimated to be between 101 and 114%, while extraction recovery was between 95 and 110% (Table 3). We applied both postcolumn infusion and spike recovery methods for the estimation of matrix effects with consistent results (postcolumn infusion: 102%, spike recovery method: 99%). A previous study estimated that approximately 20% of paracetamol in the circulation is bound to albumin independent of paracetamol level [42]. The quantitative recovery of paracetamol also contributed to the high precision of the current assay.

**Table 3.** **Process efficiency, extraction recovery and matrix effect of paracetamol in plasma (n = 3 for each condition).**

**Nominal concentration (mg/l)**	**Process efficiency mean accuracy (%)**	**Extraction recovery mean accuracy (%)**	**Matrix effect mean accuracy (%)**
0.125	101	95	106

0.25	101	110	92

5	114	108	106

30	101	109	93

Average	104	106	99

### Stability

Stability of paracetamol in plasma has been reported previously by Lou *et al*. [28], stating that paracetamol in plasma was stable for 58 days at -20°C, in the concentration range of 0.02 to 4 mg/l. Consistent with their findings, we found paracetamol to be stable upon three freeze–thaw cycles, storage at ambient condition for 4 h, and storage at 10°C for 16 h post-extraction (Table 4). The extracted samples were analyzed immediately by LC–MS/MS analysis, with autosampler kept at 4°C. Other post-extracted samples were kept at 4°C fridge before and after LC–MS/MS analysis. Therefore, extracted samples were not stored at room temperature and thus the stability at room temperature was not conducted.

**Table 4.** **Assessment of stability of paracetamol in plasma (n = 3 for each condition).**

**Nominal Concentration (mg/l)**	**3-cycle freeze-thaw**		**Auto-sample stability**		**Bench stability**	

	**Mean accuracy (%)**	**%CV**	**Mean accuracy (%)**	**%CV**	**Mean accuracy (%)**	**%CV**
0.125	100.8	2.4	99.8	3.3	98.7	1.9

0.25	99.2	1.5	97.6	1.6	99.0	4.0

5	98.3	0.5	98.6	0.9	98.8	1.4

30	97.6	0.8	97.9	1.4	98.4	0.8*

%CV: Coefficient of variation.

### Carryover

Carryover was assessed based on the relative signal of the first injection relative to the second injection, evaluated by analyzing a sample with high concentration followed by a blank injection, and then comparing the relative signal intensity observed. An injection of concentrated sample at ULOQ resulted in an insignificant signal in subsequent solvent blank, indicating negligible carryover in the current LC–MS/MS system. We calculated that significant carryover would be expected only when the concentration ratio between consecutive injections exceeded 1316-fold, which was greater than the linear range of the assay. Therefore, significant carryover only occurred if the first injection was higher than the ULOQ, or when the second injection was lower than the LLOQ. In both cases the injections were easily identified for reanalysis.

### Linearity

We evaluated different regression models according to Singtoroj *et al*. [39]. Our results showed that linear regression without weighting was the worst performing method in terms of QC predictability and calibration curve fitting (Table 5). This is expected when analyzing heteroscedastic data and can commonly be adjusted for by weighting or data transformations. Linear regression with 1/x weighting was ranked only fourth out of six models, but showed the best predictive performance of QC samples. Quadratic regression with 1/x weighting and linear regression with log–log transformation performed similarly in terms of QC precision, accuracy and calibration curve fitting. The model with the top ranking was quadratic regression with log–log transformation. Although the QC precision was ranked fourth out of six models, the accuracy of QC and calibrator fitting was the highest ranked. The finding was comparable to the work by Singtoroj *et al*. [39] in which both linear and quadratic regression with log–log transformation were the best in terms of QC and calibration curve performance among the six models. Although the commonly used linear calibration model (1/x weighting without transformation) ranked fourth in terms of overall QC and calibrator precision and accuracy, the imprecision was the lowest among the QC samples and it also provided a simple model for calibration. Therefore, the linear regression model with 1/x weight was used for quantitation purposes.

**Table 5.** **Performance of different calibration models, weights and transformations in terms of quality control and calibrator precision and accuracy.**

**Model**	**Weighting**	**Transformation**	**QC rank**		**Cal rank**	**Rank sum**	**Final**

			**Imprecision**	**%Accuracy**	**%Accuracy**		
Quadratic	None	log-log	4	1	1	6	1

Quadratic	1/X	None	2	3	2	7	2

Linear	None	log-log	3	2	2	7	2

Linear	1/X	None	1	4	4	9	4

Quadratic	None	None	6	5	5	16	5

Linear	None	None	5	6	6	17	6*

CV: Coefficient variation; QC: Quality control.

### Comparison with existing methods

In order to accurately assess the renoprotective effect of paracetamol against cell-free hemoglobin oxidative stress in malaria, the dose-response relationship must be established. While the patient response could be evaluated based on creatinine clearance, the dosage of paracetamol may not correlate with plasma concentration due to reduced drug clearance in kidney injury, comorbidity of severe malaria and reduced drug metabolism in impaired liver function. Paracetamol was known to be hepatotoxic at high concentration. Together with concomitant administration of anti-malarial drugs artesunate and artemether–lumefantrine, which inhibit cytochrome P450 enzymes, the dosage of paracetamol may not correlate with patient exposure. Therefore, the assessment of blood concentration-response relationship would be more appropriate for the purpose of the RCT study.

LC–MS/MS was chosen over ligand binding assay, which relied on the recognition of analyte by antibody and was prone to cross-reactivity and nonspecific binding. The development of the LC–MS/MS method was relatively straight-forward, and the focus was mainly on the robust sample preparation strategy from a small volume of samples and LC method with minimal matrix effect.

Upon literature review, a number of LC–MS/MS-based methodology was published by other research groups using different sample preparation and analytical approaches (Table 2). Three main approaches, namely protein precipitation, protein precipitation followed by vacuum drying and reconstitution, and the use of SPE, were used for sample preparation. Protein precipitation allowed rapid extraction of paracetamol from plasma protein. Since the process did not involve vacuum drying and SPE buffer exchange, the preparation time was the fastest among the three approaches. This was particularly suitable for the bioanalysis of large number of RCT samples for fast turnaround time.

Protein precipitation was essentially a sample dilution process, and therefore the sensitivity was usually lower compared with other sample preparation approaches. This was reflected by the LLOQ of the current method (0.125 mg/l), which was higher than other published methods. However, the imprecision of the current method was the lowest (<1.4%) since minimal preparation steps were involved. The current method required 20 μl of plasma samples, which allowed repeated analysis on limited sample volume. Compared with other methods using similar plasma volume [25,26], our methodology had a much lower imprecision (compared with approx. 7 [26] and 2.9% [25] CV). The low imprecision allowed the calculation of AUC with smaller variance. The paracetamol was administered at a high dose in the current RCT study and our data showed that only predose samples were below LLOQ. Therefore our method was fit for use in this application.

Paracetamol was freely soluble in alcohol including methanol and ethanol, and therefore methanol was considered as the mobile phase of choice during method development. Moreover, methanol also functioned as protein precipitant and internal standard solvent. Other organic solvents were considered, including acetonitrile, for protein precipitation and as the mobile phase. However, a more extensive dilution was required before LC analysis in order to achieve retention on the reverse-phase column, which may compromise the sensitivity of the assay. When acetonitrile was used as mobile phase, paracetamol was eluted close to void volume, which may also introduce matrix effect and signal suppression. The currently used methanol-based method allowed sufficient retention of paracetamol on column and achieve a reproducible matrix effect across different concentration.

## Conclusion

In this report, we presented a validated LC–MS/MS assay for the quantification of plasma paracetamol concentrations with good imprecision and accuracy, simple sample preparation using only 20 μl of plasma and short runtime. The high-throughput characteristic of the developed assay was suitable for a high volume of samples from a clinical study. By using a simple dilute-and-shoot method, it is possible to adopt the assay protocol into automated format using a liquid handler, which would further facilitate the analytical process. The assay was successfully used to quantitate plasma paracetamol concentrations in 989 samples from patients with severe and moderately severe falciparum malaria.

## Future perspective

We anticipated that the sample volume requirement, assay imprecision, inaccuracy and specificity will be further improved together with technology advancement. While LC–MS/MS has the advantage of analytical specificity, the analysis throughput was limited by the sample preparation procedure, which still required manual handling. In the future, we anticipate more research and development in the direction of automation, including automated liquid handling for sample preparation, or primary tube sampling coupled with on-line sample extraction.

**Summary points**

- Rapid, simple and precise LC–MS/MS paracetamol quantification with less than 6-min run time and small volume (20 μl) of plasma required.
- Low imprecision compared with existing methodologies, minimum matrix effect across different concentration and minimum carryover between injections.
- Fully validated and high-throughput method used as part of randomized clinical trial.
